# Probing the local environment of a single OPE3 molecule using inelastic tunneling electron spectroscopy

**DOI:** 10.3762/bjnano.6.257

**Published:** 2015-12-24

**Authors:** Riccardo Frisenda, Mickael L Perrin, Herre S J van der Zant

**Affiliations:** 1 Kavli Institute of Nanoscience, Delft University of Technology, Lorentzweg 1, 2628 CJ Delft, Netherlands

**Keywords:** current–voltage characteristics, DFT calculations, mechanically controllable break junction (MCBJ), molecule–electrode interaction, vibrational modes

## Abstract

We study single-molecule oligo(phenylene ethynylene)dithiol junctions by means of inelastic electron tunneling spectroscopy (IETS). The molecule is contacted with gold nano-electrodes formed with the mechanically controllable break junction technique. We record the IETS spectrum of the molecule from direct current measurements, both as a function of time and electrode separation. We find that for fixed electrode separation the molecule switches between various configurations, which are characterized by different IETS spectra. Similar variations in the IETS signal are observed during atomic rearrangements upon stretching of the molecular junction. Using quantum chemistry calculations, we identity some of the vibrational modes which constitute a chemical fingerprint of the molecule. In addition, changes can be attributed to rearrangements of the local molecular environment, in particular at the molecule–electrode interface. This study shows the importance of taking into account the interaction with the electrodes when describing inelastic contributions to transport through single-molecule junctions.

## Introduction

Vibrational degrees of freedom in molecules are of crucial importance in many physical, chemical and biological processes [1–2]. In recent years, their involvement in biological processes has attracted much attention, for instance in the olfactory system [3–5] and in photosynthetic activity of chromophores [6–7]. In these systems the processes occurring at the single-molecule level are dramatically influenced by the environment. Therefore, to understand the role of vibrations, experiments that can study the properties at the single-molecule level are very suited [8], as they do not suffer from collective effects and ensemble averaging. Different approaches have been proposed to extract the vibrational spectrum of an individual molecule, either employing optical [9–11] or electrical [12–15] measurements. Among the electrical methods, many different approaches have been employed such as current fluctuations [14], resonant transport [16–17], and inelastic electron tunneling spectroscopy (IETS) [12–13,18–21], of which the latter is the most popular.

**Figure 1:**
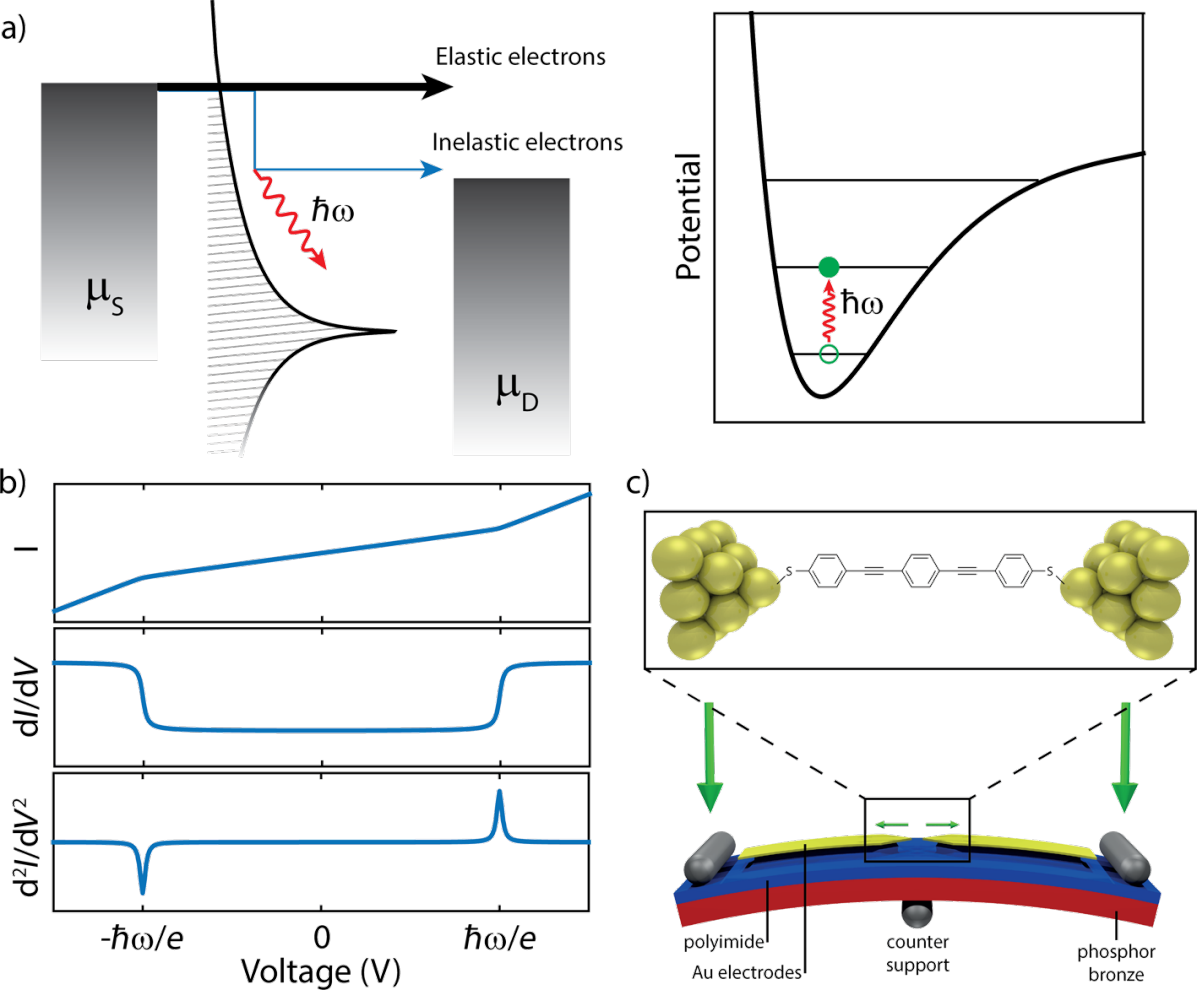
(a) Schematic of the inelastic electron tunneling spectroscopy (IETS) in a single-molecule junction. Charge transport happens through the tail of the Lorentzian broadened occupied molecular level depicted. The wiggly red line represents a molecular vibration excited after an inelastic scattering event (shown also in the right panel). (b) IETS shows up as a kink in the *IV* (upper panel), a step in the d*I*/d*V* (middle panel), and a peak in d^2^*I*/d*V*^2^ (lower panel). (c) Schematic illustration of the MCBJ technique. The large green arrow indicate the force to bend the sample. The small arrows illustrate the attenuated electrode displacement. The inset shows an idealized illustration of a single-molecule junction, with OPE3 trapped between the electrodes.

Figure 1a schematically depicts the IETS process, where the metallic electrodes are represented as Fermi distributions. In between these electrodes a molecule resides which is described as a Lorentzian broadened single level coupled to a vibrational mode. The molecular level is located outside the bias window, and at low bias voltage, transport via this level is elastic and off-resonant. The elastic contribution results in an approximately linear dependence of the current on the voltage, as can be seen in the upper panel of Figure 1b. When the bias voltage is larger than the vibrational mode energy 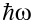/e, an electron from the left lead can tunnel inelastically to the right lead by exciting a vibration of the molecule. In this process, the electron loses an energy 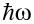, and a phonon with the same energy is created. In the case of a small inelastic current, the vibrationally excited molecule then returns to its ground state typically before the excitation of a new vibration by a subsequent electron. The excess energy is converted to phonons in the electrodes and/or the formation of an electron–hole pair [22]. This inelastic contribution of the current leads to a kink in the current–voltage characteristic (*IV*) at the vibrational energy, as shown in Figure 1b. However, as the ratio between the elastic and inelastic currents is large, the vibrational excitations become more evident when looking at the differential conductance (d*I*/d*V*) or the d^2^*I*/d*V*^2^, where they show up as steps, or peaks (dips), respectively. In this manuscript, when dealing with experimental data, we call IETS spectra the d^2^*I*/d*V*^2^/(d*I*/d*V*) signals calculated from the *IV* characteristics.

Here, we investigate the IETS signal of an oligo(phenylene ethynylene)dithiol molecule (OPE3) single-molecule junction at liquid helium temperature (4.2 K). Exploiting the high stability of the mechanically controlled break junction technique in cryogenic vacuum, we investigate the evolution of the junction in time. Multiple junction configurations are observed with distinct IETS spectra. In a second experiment, we monitor the IETS spectrum of a molecular junction as a function of the electrode displacement. The IETS spectra recorded at different locations display large variations among each other. A comparison with quantum chemistry calculations for different junction geometries allows for identifying some of the vibrational modes contributing to transport. Our findings suggest that the different junction configurations are characterized by differences in the molecule–electrode interface.

## Results

**Figure 2:**
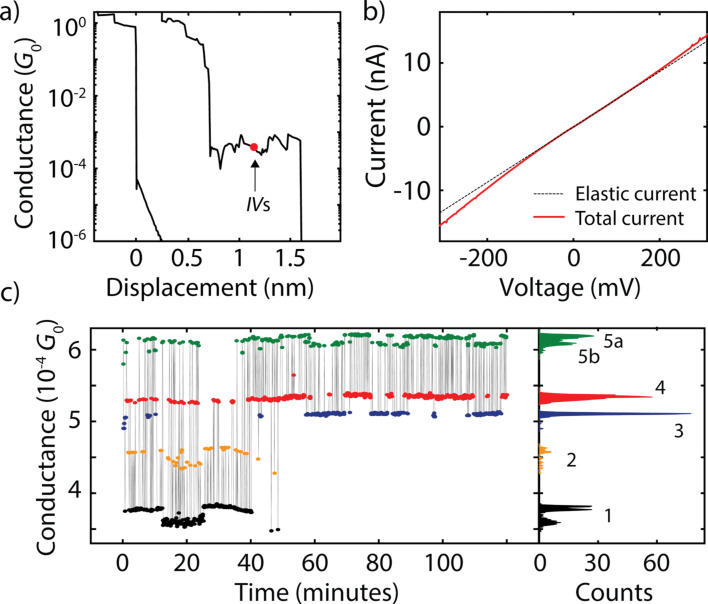
(a) Typical breaking traces recorded on a junction without molecule (left) and with OPE3 (right). (b) IV recorded at the location indicated by a red dot along the OPE3 plateau in (a). The dashed line represents the elastic contribution obtained from a linear fit at low-bias. (c) Low-bias conductance for the 1200 consecutive *IV*s recorded at the location indicated by a red dot along the OPE3 plateau in (a). The colors used in (c) correspond to the different junction configurations 1–5.

In our implementation of the MCBJ technique, a lithographically defined gold constriction is broken by bending the substrate in a three-point bending mechanism. Upon rupture of the gold contact, two atomically sharp electrodes are formed, of which the separation can be tuned with picometer resolution. When breaking the gold wire in the presence of OPE3, a molecule can bridge the freshly broken electrodes, as is schematically illustrated in Figure 1c. When measuring the conductance as a function of electrode displacement (a so-called breaking trace), one can distinguish two types of behavior. The left breaking trace in Figure 2a shows the typical signal of an empty junction, in which the conductance after the rupture of the last gold contact decreases exponentially with distance. This is a signature of vacuum tunneling between two metallic electrodes. Once a molecular junction is formed (junction 1, right breaking trace in Figure 2a, a plateau in the conductance is observed, in this particular case around 5·10^−4^*G*_0_ a value that matches well previous studies on OPE3 dithiol molecular junctions [23]. By increasing the distance between the electrodes, one can eventually break the molecular junction, characterized by an abrupt drop in conductance. The high mechanical stability of the electrodes at low-temperature allows to interrupt the stretching while being on a plateau, and perform *IV* measurements.

Figure 2b shows an *IV* recorded at the position indicated by the red dot in Figure 2a. The current is linear at low bias due to the dominating elastic contribution, which is indicated by the dashed line. Above a certain voltage threshold, the current deviates as a result of an additional inelastic contribution. When numerically calculating the second derivative, peaks and dips are visible. We recorded 1200 consecutive *IV*s for the same fixed electrode position. For all these *IV*s, the low-bias conductance is calculated using a linear fit, and shown in Figure 2c as a function of time. The conductance fluctuates between 3.5·10^−4^ and 6.5·10^−4^*G*_0_. Remarkably, the conductance clusters around specific values, suggesting the presence of multiple junction configurations at the same electrode separation, between which the molecule switches. About seven to eight configurations can be distinguished. To identify these, we build a conductance histogram (see right panel), in which peaks represent the most probable conductance values.

**Figure 3:**
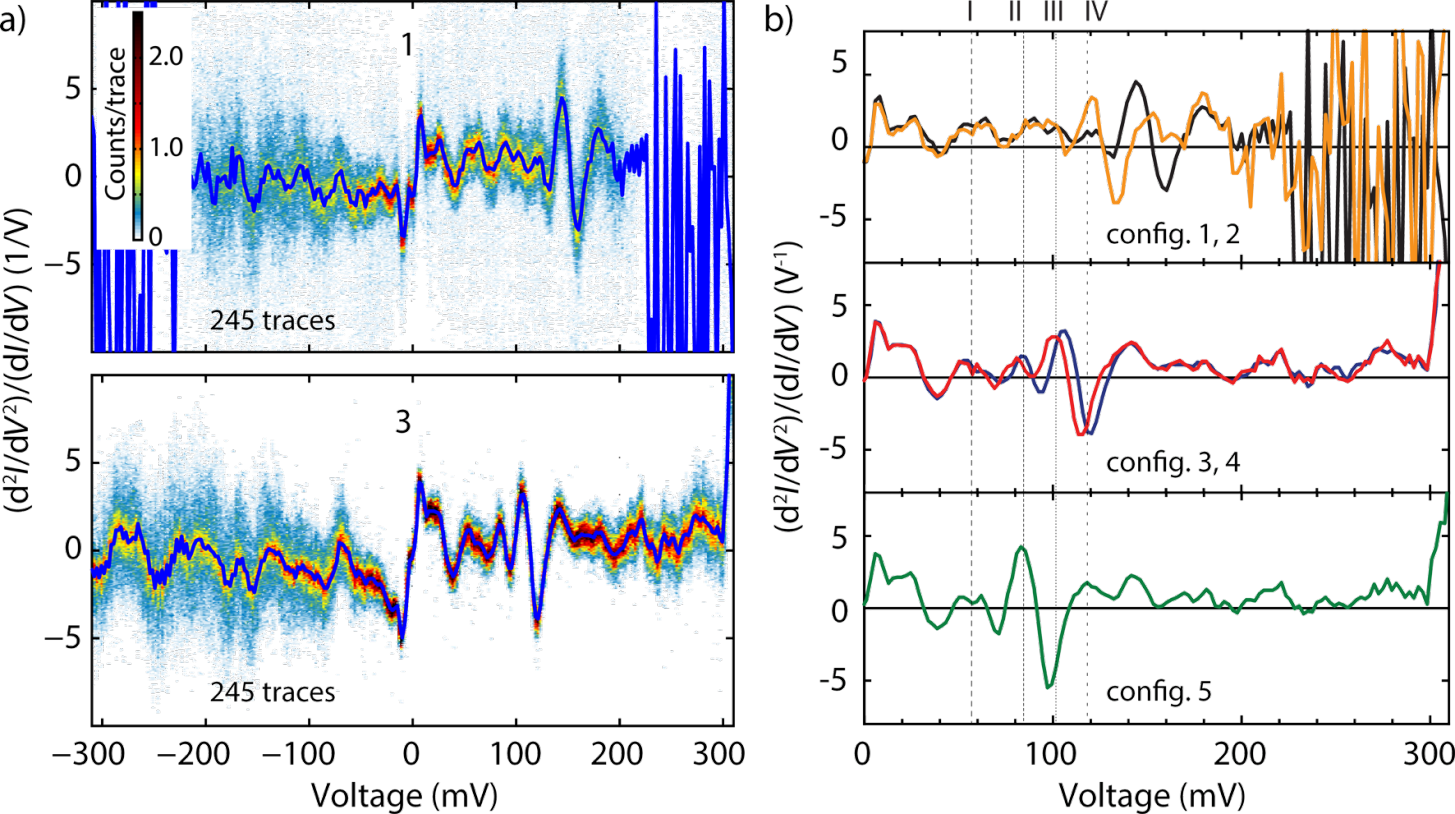
(a) Two-dimensional histograms of two configurations built from the individual spectra measured on OPE3. The histograms are built from all the individual second derivative traces by binning the x-axis with 600 bins/V and the y-axis with 12 bins/(1/V). (b) Master-curves of the spectra for the five junction configurations extracted from the two-dimensional histograms. The colors used in (b) correspond to the different junction configurations 1–5.

To gain more insight in the inelastic contributions to transport, we extracted the IETS spectra for the different junction configurations. Since the ratio between the inelastic and elastic contributions is small and bias-induced fluctuations are present [24], we employ a novel method to extract a reliable IETS signal. In our approach, we record a large number of *IV*s, from which we calculate the first and the second derivative. We then divide point by point the second derivative by the first derivative to obtain the IETS signal. To increase the signal-to-noise ratio and reduce the effect of fluctuations, we build two-dimensional histogram from all the individual IETS curves of which two examples are shown in Figure 3a. An IETS ‘master-curve’ signal is constructed by extracting the most probable IETS value at each bias point using a Gaussian fit. For more details about our approach, see Supporting Information File 1.

Our extracted IETS spectra exhibit both peaks and dips and a summary of the observed (antisymmetric) peak energies is presented in Table 1; the table only contains energies of peaks for which a dip at the corresponding negative bias has been observed (see Figure S3 of Supporting Information File 1). As discussed in the introduction, from theoretical considerations, vibrations show up in the IETS spectra as peak/dip pairs which should be anti-symmetric in the applied bias voltage. In the experiment however, not all peaks are anti-symmetric and for some the line shape appears to be asymmetric in bias. In literature, the lack of anti-symmetry in a IETS spectrum can be explained by an asymmetry in the contacts [25–26], leading to a shift in the peaks energy or a change in the amplitude for different bias polarities. Another possibility is that some of the experimental peaks/dips do not have a vibrational origin. This issue deserves further studies, both experimentally and theoretically.

**Table 1:** Experimental IETS peaks energies (junction 1) and theoretical vibrational modes.

exp. (mV)	DFT (meV)	description	notes

24–27	26	Au–S	
58–59	59	entire molecule	
80–85	80	in-plane ring	I
105	102	in-plane ring	II
119–122	126	in-plane ring	III
—	130	C–S	IV
180–183	183	in-plane ring	
215–220	—		
275–280	270	C≡C	

In the remainder of the paper. we will discuss only peaks that can both be found at the negative and positive biases within 5 mV. In Figure 3b we show the IETS spectrum for the junction configuration 1–5 at positive bias voltages. In all five configurations peaks around 25 and 60 mV are present. For bias voltages between 60 and 140 mV, four peaks are visible (numbered I–IV in Figure 3b), of which the position and amplitude depend on the junction configuration. In addition, a few dips are present. In the bias range of 140 to 300 mV, all junctions show a peak around 180 mV. Junction 1–2 then show a strong noise for biases larger than 200 mV. For junction 3–5, this noise is not as pronounced and peaks can be distinguished at 220 and 275 mV.

**Figure 4:**
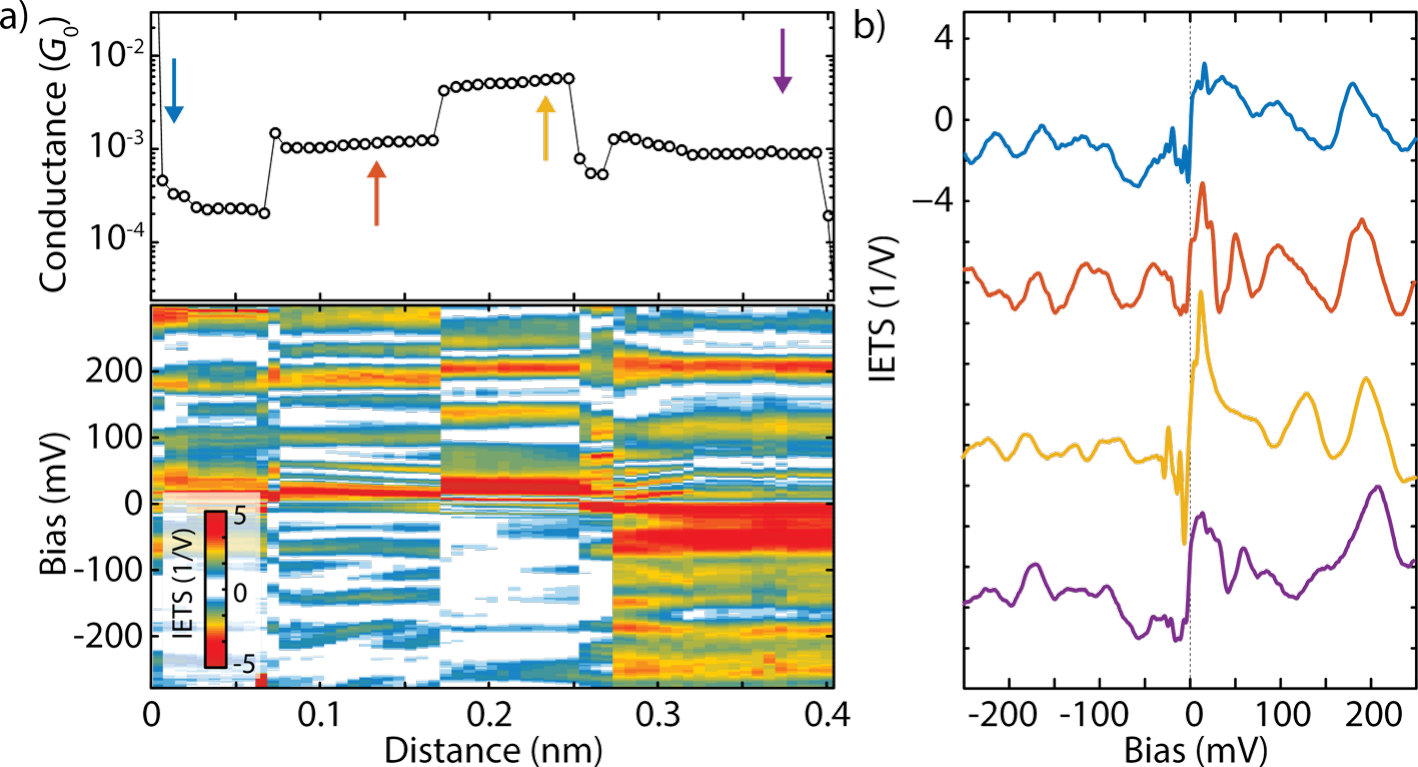
(a) Low-bias conductance trace recorded during the stretching of an OPE3 single-molecule junction (top) and color-map representing the IETS signal upon stretching (bottom). (b) Extracted IETS master-curves at the positions indicated by the arrows in (a). Note that not all peaks/steps are visible in the color-map for this choice of contrast.

In the measurements presented previously, different IETS spectra were recorded due to switches between different configurations, while the electrode separation was being kept constant. In the following, we investigate the influence of the displacement on the IETS spectra on a different single-molecule junction (junction 2). We started from the metallic regime (conductance *>* 20*G*_0_) and after having broken the gold quantum point contact we formed a molecular junction. During the stretching of this junction, for each electrode position, 250 *IV*s were recorded and the IETS master-curve was extracted. In between two IETS spectra, the electrode separation was increased by 6 pm. The top panel of Figure 4a shows the conductance breaking trace extracted from a linear fit of the *IV*s at low bias. The plateau in conductance indicates the formation of the single-molecule junction. Continuous regions are observed and separated by steps, which are attributed to stress-releasing rearrangements of the molecular junction.

In the lower panel of Figure 4a we present the IETS spectra as a function of stretching as a color map. Peaks and dips are present in the IETS spectra, as observed previously. Along the continuous conductance regions, the features evolve smoothly with small changes in position and amplitude. In contrast, large differences are present between different regions, with pronounced rearrangements of the peaks and the dips. This becomes evident from Figure 4b, in which one spectrum is presented for each continuous conductance region. We note that for the second and fourth region, the low-bias conductance is similar, but the IETS spectra are different. This shows that junctions with similar low-bias conductance can have different IETS spectra and overall, the measurement point to a large dependence of the IETS features on the local environment. A similar conclusion has been drawn by Ward et al. for the Raman response of single-molecule junctions [27], in which spectral diffusion and blinking were reported.

**Figure 5:**
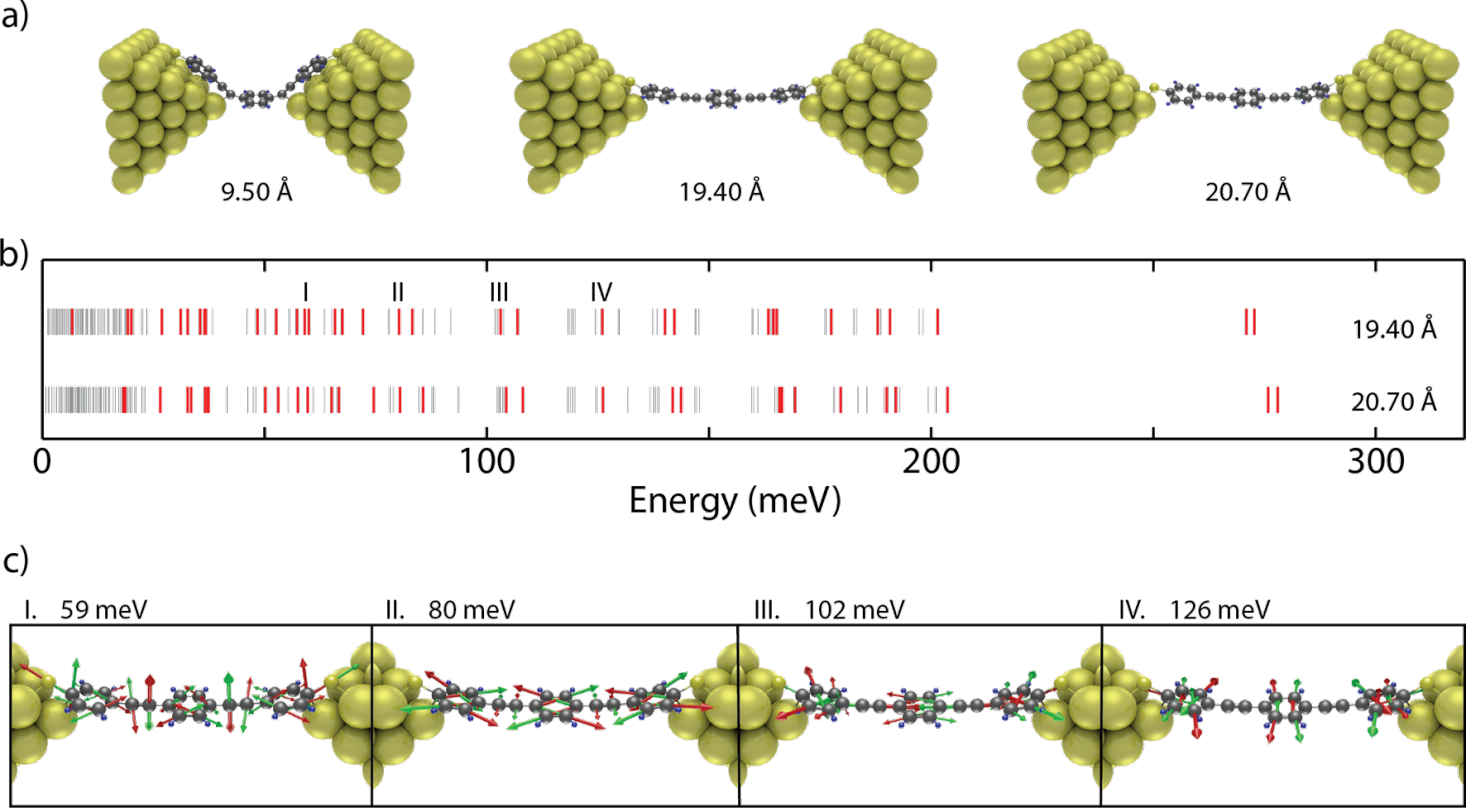
(a) Three selected geometries along the stretching of the junction. (b) Calculated vibrational spectra for an electrode displacement of 19.40 Å (top) and 20.70 Å (bottom). (c) Vibrational modes responsible for the peaks I–IV in (b).

To relate the peaks in the IETS spectra to the vibrational modes, we used the Amsterdam Density Functional (ADF) package and performed density function theory (DFT) calculations of the OPE3 molecular junction. All calculations were optimized using a TZP Slater-type orbital local basis-set and the PBE GGA functional. We stretch the molecular junction starting from the configuration shown in the left panel of Figure 5a (for more details see Supporting Information File 1). During the stretching of the junction, we observe continuous regions where the stress accumulates (see Figure S4 of Supporting Information File 1), separated by events in which the molecule typically switches between binding sites on the electrodes. This can explain the switches in the conductance plateau of Figure 4a and, as we have seen, the IETS spectrum show large variation before and after the jump.

The middle and right panel of Figure 5a show two typical geometries obtained during the stretching. In the first geometry (19.40 Å), in addition to the usual Au–S covalent bond, the molecule interacts on both sides with the outer phenyl rings. In the second geometry (20.70 Å), the molecule is more extended and interacts only on one side with the outer ring, while on the other side the binding occurs through the sulphur atoms. For the two geometries we calculated the frequency spectrum, as shown with lines in Figure 5b. Some of those modes are expected to couple to transport. Due to the lack of selection rules (in contrast to infrared and Raman scattering processes), we highlight in red the modes that are expected to contribute to transport, based on the propensity rules proposed by Troisi and Ratner [28]. In the OPE3 spectrum, many modes are expected to couple to transport due to their symmetry and their modulation of the π-system. Comparing the two geometries, we notice that for the largest separation, some modes shift towards higher energies, an indication of the stretching of the molecule.

Comparing the experimental modes with the calculated energies, the following observations can be made: some of the vibrational modes (183 and 270 meV) correspond to modes reported in literature [28–29] and are known to couple to transport. These originate from the phenyl rings and the ethynyl (C≡C) triple bonds, respectively. In the experiments, we observe peaks in the spectra around the energies of those modes. The peak around 180 mV is observed in all configurations, and attributed to vibrational modes involving the center and outer phenyl rings. In configuration 1–2, noise appears for bias voltage larger that 220 mV and seems to correspond with the onset of an additional contribution to the noise. In configuration 3–5, this noise contribution is less pronounced and a peak around 275 mV is observed, which matches the calculated mode of the stretching of the ethynyl bond (C≡C). We also note the presence in all configurations of a peak around 25 meV, which corresponds to vibrations involving the Au–S bonds. The modes discussed above constitute a chemical fingerprint of the molecule in the junction, and are consistent with previous experiments on large-area OPE3 junctions [29] and theoretical predictions [30–31]. We would like to stress that, although we attributed each peak to a single vibrational mode, in the experiments, the peaks may originate from multiple modes that are located closely together, as can be seen in the DFT spectrum. The peak at 215–220 mV is not identified in the calculations. This may be due to contaminants close to the molecular junction, and/or overtones of lower energy modes.

In contrast to the modes mentioned above, the experimental modes labeled with Roman numbers in Table 1 and Figure 2 are present with different intensities and/or different energies. These modes are located between 80 and 120 meV and interestingly, they are mostly related to in-plane ring modes. This could indicate that one or more phenyl rings interact differently with the metallic electrode and that the molecule–metal interface plays a large role in determining the IETS spectra. Thus, next to constituting the chemical fingerprint of the molecule, the IETS spectra contain additional information about the molecular geometry in the junction. Altogether, the changes in the spectra may be related to small changes in the molecular geometry at the interface between the molecule and the metallic electrodes or in the electronic configuration.

**Figure 6:**
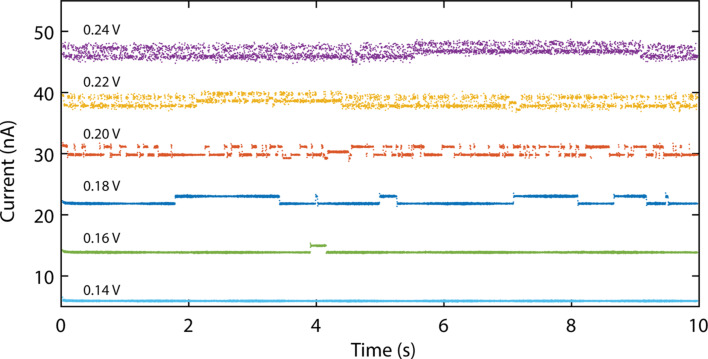
Current versus time traces acquired at bias voltages varying between 0.14 and 0.24 V. The traces have been offset vertically by 7 nA for clarity. Notice the increase in switching rate for higher bias voltages.

Finally, to investigate the switching dynamics of OPE3 between the different configurations (1–5) we recorded the conductance of junction 1 as a function of time (see Figure 6a). The traces have been acquired at a rate of 400 Hz for bias voltages ranging from 0.14 to 0.24 V. For clarity, the consecutive traces have been offset by 7 nA. For bias voltages below 0.14 V, the current does not change over time, for example at 0.14 V the average current is 2.4 nA. At 0.16 V, a switch is observed around 4 s to a higher value and then it switches back to the original values shortly thereafter. For increasing voltage, the switching behavior becomes more frequent, resembling telegraph noise. This points to a two-level system characterized by two different conductance values [32]. By comparing the conductance values with the different configurations obtained in Figure 2c, we identify the low-conductance state as configuration 3 and the high-conductance state as 5a. Following the reasoning obtained from the IETS spectra, the two-level fluctuations may be caused by the different configurations. Finally, we notice that for bias voltages higher than 0.20 V, some of the two-level fluctuations involve configurations 4 and 5b, and four levels of current are present. In conclusion we observed fluctuations induced by the bias voltage, two-level switching between 3 and 5a, above 0.16 V, and four-level switching between 3, 4, 5a and 5b, above 0.22 V.

## Conclusion

In conclusion, we studied the IETS spectra of OPE3 single-molecule junctions, both as a function of time and electrode separation. We find that the IETS spectra depend heavily on the junction geometry and are sensitive to the local molecular environment. We compared our results to quantum chemistry calculations for the molecule sandwiched between gold electrodes and identified some of the peaks in the experimental spectra. Finally, current versus time traces for different bias voltages reveal an interesting interplay between bias voltage and current fluctuations caused by different molecular configurations. Our findings provide a way to gain additional information regarding the molecule–electrode interaction, in particular, the interesting interplay between molecular conformation, vibrations and charge transport.

## Supporting Information

File 1 Additional experimental data.
