# Supplementary material for: Probing the local environment of a single OPE3 molecule using inelastic tunneling electron spectroscopy
Source: Beilstein J Nanotechnol. 2015 Dec 24;6:2477–84. doi: 10.3762/bjnano.6.257 (PMC4734430; doi:10.3762/bjnano.6.257)
Supplement: File 1 — Additional experimental data. [file Beilstein_J_Nanotechnol-06-2477-s001.pdf]

Supporting Information

for

Probing the local environment of a single OPE3 molecule using inelastic electron tunneling spectroscopy

Riccardo Frisenda[1], Mickael L. Perrin[1] and Herre S. J. van der Zant*[1]

Address: [1]Kavli Institute of Nanoscience, Delft University of Technology, Lorentzweg 1, 2628 CJ Delft, Netherlands

Email: Herre S. J. van der Zant - H.S.J.vanderZant@tudelft.nl

* Corresponding author

Additional experimental data

IETS

The IETS spectra of Figure 3 and Figure 4 of the main text have been extracted from direct current versus voltage measurements. The inelastic contribution in a single molecule is a small signal that is best visualized in the first and second derivative of the current with respect to the voltage. To measure such signals, lock-in techniques are usually employed, because of the high sensitivity and the possibility of recording directly the first and/or second derivative of the current. Apart from the small signal, another challenge present in single-molecule IETS measurements is the eventual presence of fluctuations in the current (thermal- and/or voltage-activated). The interplay between the nuclear and electronic degrees of freedom makes the presence of noise in the current a common occurrence in single-molecule junctions [1]. The noise in the current is amplified when numerically calculating the IETS spectrum because of the first and second derivative operations. This problem makes the extraction of a reliable IETS signal in a noisy junction a challenging task, even employing a lock-in technique. To overcome the challenge of small inelastic signals, one can improve the signal-to-noise ratio by measuring a large number of samples for every bias voltage point and then averaging the current. The problem of fluctuations in the signal can be solved by acquiring the data faster than the typical timescale at which the fluctuations occur. The solution for both problems are opposite, since they require either a slow or a fast measurement.

We propose a novel method to extract a reliable IETS signal from direct current versus voltage measurements of noisy single-molecule junctions. Our strategy is based on the acquisition of a large number of consecutive IVs, as shown in Figure S1. We sweep the bias voltage at a rate of 200 mV/s and sample the current at 6400 Hz with a linear amplifier (gain 10^8 V/A). The bias voltage resolution is 0.305 mV and the time that it takes to measure one IV is about 6 s. We extract the IETS signal from each IV by performing numerical smoothing and differentiation, dividing the second derivative of the current by the first derivative of the current point by point. We then build a two-dimensional histogram from all the individual IETS curves by binning at the same time the bias voltage on the x-axis and the IETS signal on the y-axis, as shown in Figure S2a for the five configurations. To

extract the IETS 'master-curve', we find the maximum of the histogram for each bias point using a Gaussian fit (see the line cuts in Figure S2b).

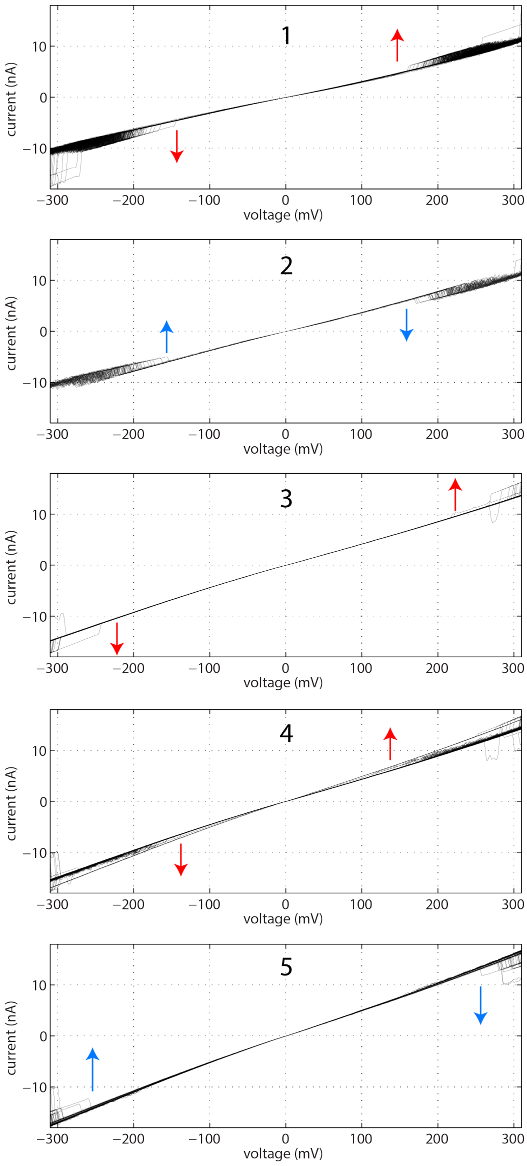

Figure S1: Current–voltage characteristics of configurations 1–5 of junction 1. In total we recorded 1200 consecutive IVs. The arrows indicate the voltage onset of noise/fluctuations in the current. The direction of the arrows indicates whether the conductance increases or decreases during the fluctuations.

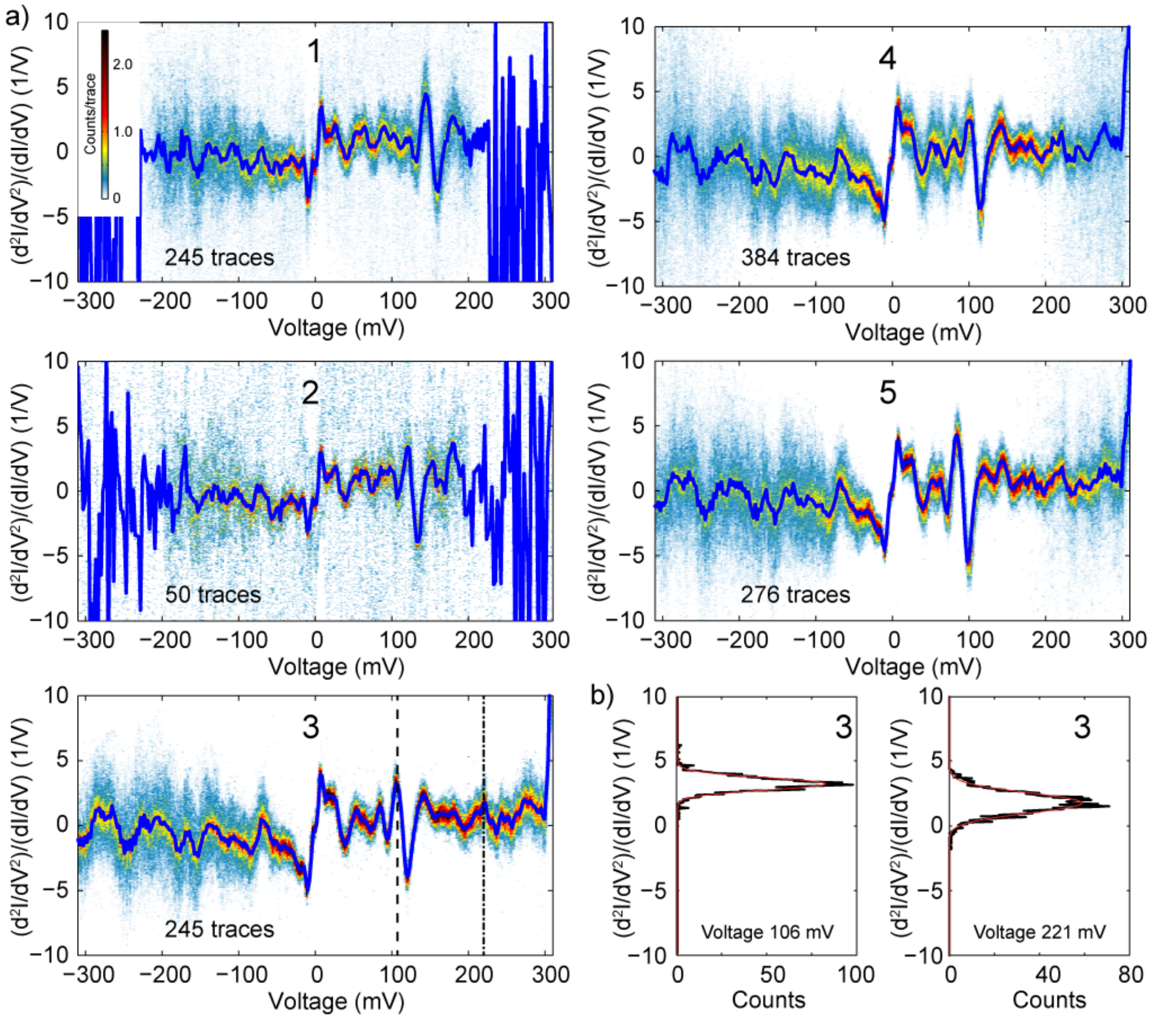

Figure S2: Two-dimensional histograms of the five configurations built from all the individual 1200 IETS spectra measured on junction 1. (a) The measured data have been divided in five categories according to the low-bias conductance as detailed in the main text. The histograms are built from all the individual IETS traces by binning the bias voltage with 600 bins/V and the IETS with 12 bins/(1/V) (b) Line cut obtained on junction 3, including the Gaussian fit used to extract the maximum of the IETS signal, for two bias voltages.

The advantage of this method is its robustness, even for noisy junctions. To illustrate this, in configurations 3–5, current fluctuations are prominent, as is observed in Figure S1. However, when extracting the master curve using our novel method, it is not affected by those fluctuations. For configurations 1 and 2, the limited amount of IVs (as can be seen in Figure 2 of the main text, configurations 1 and 2 are only accessed in the first part of the experiment, resulting in a limited

amount of IVs), renders this method unsuccessful above 200 mV. At this voltage, the noise is too high and the extracted master IETS signal becomes unreliable.

Figure S3 shows the IETS master-curve of each of the five configurations. The blue curves correspond to the normal signal, while the red curves are the IETS signal mirror in respect to both the x-axis and y-axis. In this way one can more easiliy visualize the anti-symmetric peak/dip pairs.

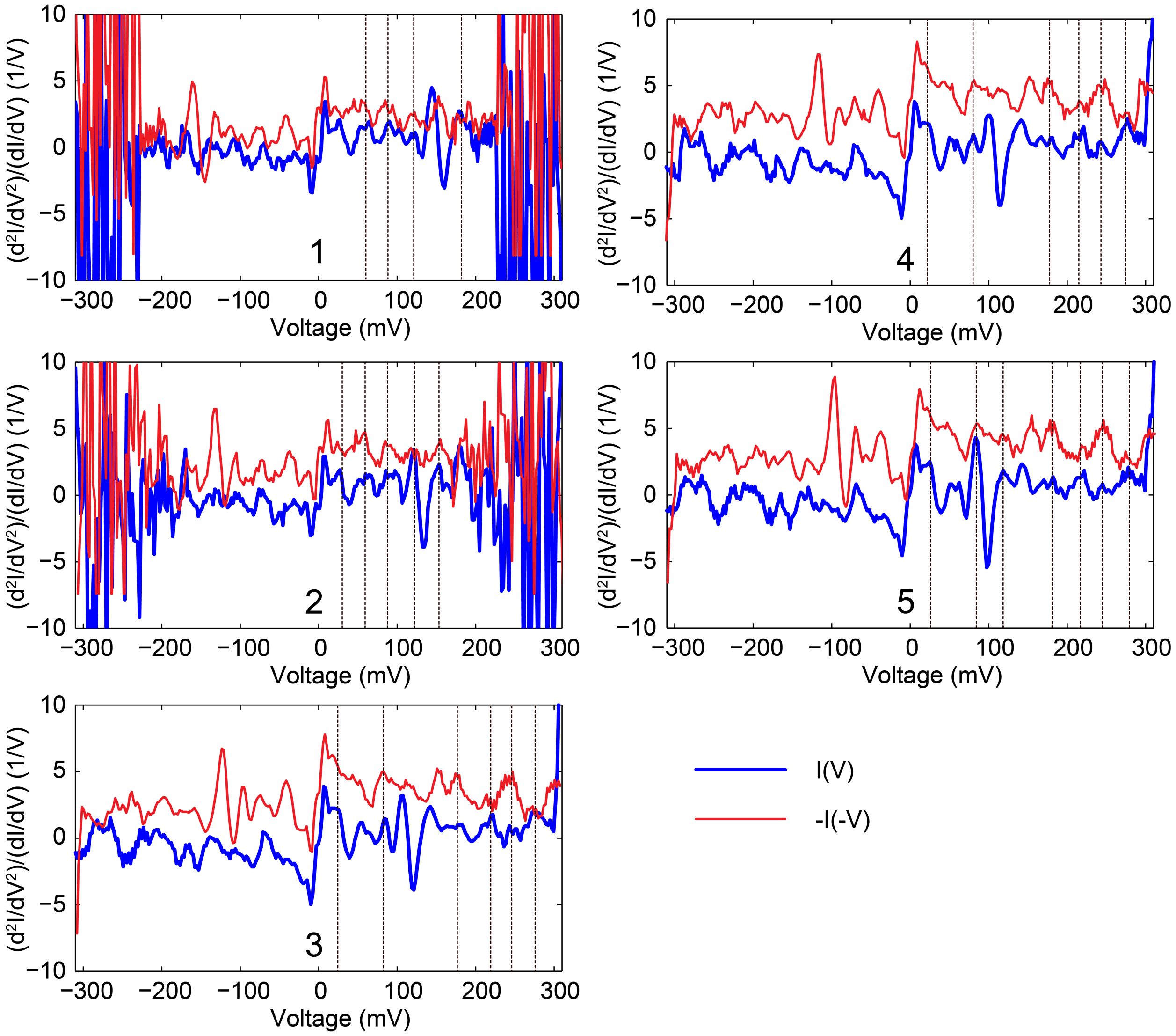

Figure S3: IETS master-curves of the five configurations of junction 1. The red and blue curves represent, respectively, the IETS master-curve (blue) and the same curve but with current and bias axis reversed (red). The dashed black lines indicate the anti-symmetric peaks in the spectra.

DFT + NEGF calculations OPE3 stretching

Density functional theory (DFT) calculations were performed using the Amsterdam Density Functional (ADF) package [2,3], using the GGA PBE exchange-correlation functional and the triple-ζ plus polarization (TZP), Slater-type orbital local basis-set. To account for relativistic effects in the electrodes, the zeroth order regular approximation (ZORA) to the Dirac equation was used. Geometries were converged to energy changes of less than 10^{-3} hartree, energy gradients of less than $1 \cdot 10^{-3}$ hartree/Å maximum and $6.7 \cdot 10^{-4}$ hartree/Å RMS.

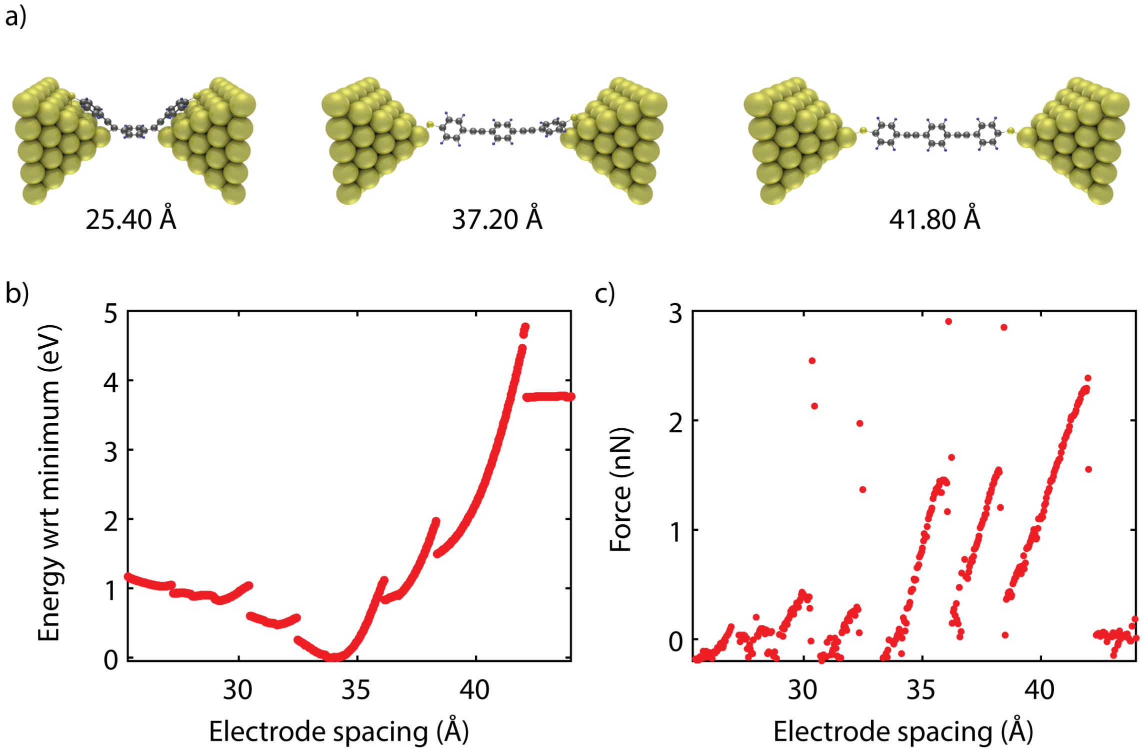

Figure S4: DFT calculations while stretching a OPE3 molecular junction. (a) Junction geometries for three different electrode separation during stretching. (b) Total energy of the system during stretching. (c) Magnitude of the force along the stretching direction.

The electrode were modeled as two gold pyramids terminated with (111) surfaces. Each electrode consists of 5 layers with 55 atoms each, as shown in Figure S4. The initial electrode separation is 25.40 Å, as shown in the left panel. The electrode separation is determined from the distance between the outer gold layers, center-to-center. The calculations were performed as follows. First,

the molecular geometry was relaxed for the electrode while keeping the coordinates of the outer three gold layers fixed. Only the inner two were allowed to relax. After convergence, the Au-Au distance was varied from 25.40 to 44.00 Å in steps of 0.04 Å. For every step, the geometry was relaxed. Vibrational modes calculations were performed analytically within ADF[2,3], including the gold atoms of the electrodes.

Vibrational modes

For the geometry with an electrode separation of 19.10 Å shown in the main text, Figure S5 presents a collection of vibrational modes.

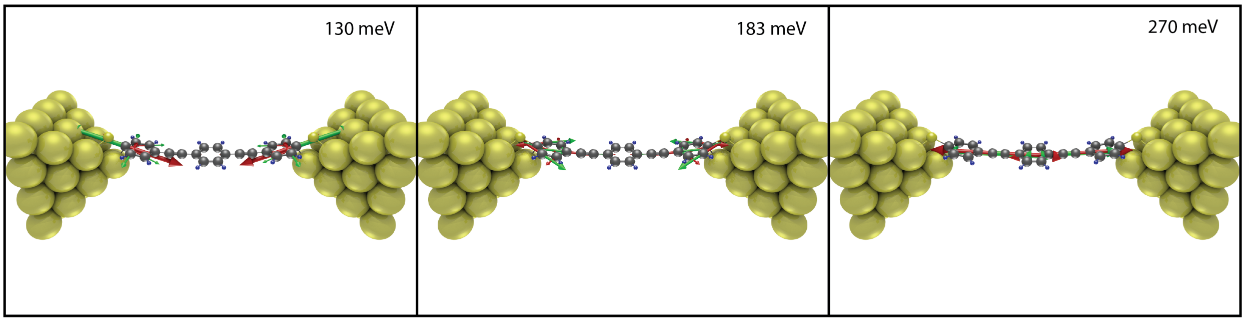

Figure S5: Three selected vibrational modes. The size of the arrows indicate the nuclear displacement amplitudes. Arrows of the same color move in phase with each other.

References

1. Secker, D.; Wagner, S.; Ballmann, S.; Härtle, R.; Thoss, M.; Weber, H. B. *Phys. Rev. Lett.* **2011**, *106*, 136807. doi:10.1103/PhysRevLett.106.136807.

2. Fonseca Guerra, C.; Snijders, J. G.; te Velde, G.; Baerends, E. J. *Theor. Chem. Acc.* **1998**, *99*, 391–403. doi:10.1007/s002140050353.

3. te Velde, G.; Bickelhaupt, F. M.; van Gisbergen, S. J. A.; Fonseca Guerra, C.; Baerends, E. J.; Snijders, J. G.; Ziegler, T. *J. Comput. Chem.* **2001**, *22*, 931–967. doi:10.1002/jcc.1056.
